# Nest Site Selection during Colony Relocation in Yucatan Peninsula Populations of the Ponerine Ants *Neoponera villosa* (Hymenoptera: Formicidae)

**DOI:** 10.3390/insects11030200

**Published:** 2020-03-23

**Authors:** Franklin H. Rocha, Jean-Paul Lachaud, Yann Hénaut, Carmen Pozo, Gabriela Pérez-Lachaud

**Affiliations:** 1 El Colegio de la Frontera Sur, Conservación de la Biodiversidad, Avenida Centenario km 5.5, Chetumal 77014, Quintana Roo, Mexico; frocha.vela@gmail.com (F.H.R.); jlachaud@ecosur.mx (J.-P.L.); yhenaut@ecosur.mx (Y.H.); cpozo@ecosur.mx (C.P.); 2 Centre de Recherches sur la Cognition Animale (CRCA), Centre de Biologie Intégrative (CBI), Université de Toulouse; CNRS, UPS, 31062 Toulouse, France

**Keywords:** *Aechmea bracteata*, bromeliad, Ponerinae, tandem running, colony relocation, adaptation

## Abstract

In the Yucatan Peninsula, the ponerine ant *Neoponera villosa* nests almost exclusively in tank bromeliads, *Aechmea bracteata.* In this study, we aimed to determine the factors influencing nest site selection during nest relocation which is regularly promoted by hurricanes in this area. Using ants with and without previous experience of *Ae. bracteata*, we tested their preference for refuges consisting of *Ae. bracteata* leaves over two other bromeliads, *Ae. bromeliifolia* and *Ananas comosus*. We further evaluated bromeliad-associated traits that could influence nest site selection (form and size). Workers with and without previous contact with *Ae. bracteata* significantly preferred this species over others, suggesting the existence of an innate attraction to this bromeliad. However, preference was not influenced by previous contact with *Ae. bracteata*. Workers easily discriminated between shelters of *Ae. bracteata* and *A. comosus*, but not those of the closely related *Ae. bromeliifolia*. In marked contrast, ants discriminated between similar sized *Ae. bracteata* and *Ae. bromeliifolia* plants, suggesting that chemical cues and plant structure play an important role. Size was also significant as they selected the largest plant when provided two dissimilar *Ae. bracteata* plants. Nest site selection by *N. villosa* workers seems to depend on innate preferences but familiarization with plant stimuli is not excluded.

## 1. Introduction

Many species of social hymenoptera frequently move to new nests sites, although emigration presents significant challenges and risks [1,2], and often implies a fitness cost [3,4]. Colony relocation is a common phenomenon in ants [1,5]. Some ant species move their nests as part of their life history (e.g., army ants), but the majority do so in response to numerous biotic and abiotic factors, including microclimate fluctuation [6,7,8], physical disturbance [9,10], intra- and interspecific competition [1,4,11,12,13], resource availability [14,15], and predator or parasite pressure [5,12,14,16]. Arboreal ants are particularly prone to move their colonies from one site to another [1] as occurs commonly in the Neotropical ponerine ant, *Neoponera villosa* (Fabricius) (Hymenoptera: Formicidae) [17]. 

*Neoponera villosa* is a generalist arboreal predatory ant [18,19,20] with a wide geographical distribution, from Texas to Argentina [21]. This species occurs both in wet and dry forests [22] and is an opportunistic cavity breeder that nests in dead and live trees, and in bromeliads [17,23,24]. In the southern part of the Yucatan Peninsula, Mexico, *N. villosa* nests mainly in the epiphytic bromeliad *Aechmea bracteata* (Sw.) Griseb [23,25,26], although other species of *Aechmea* with the same type of growth are available in this area [27]. Workers measure 12 to 13 mm [28] and colonies nesting in *Ae. bracteata* contain 97.8 ± 7.9 workers (mean ± SEM, n = 82, range 3–322) [29]. *Aechmea bracteata* is a "phytotelm tank" type bromeliad and mature plants present a waterproof central cavity suitable for housing ants [23,26]; large groups of shoots at different stages of maturity develop from a rhizome [30]. This bromeliad is characteristic of the inundated forest of the Sian Ka’an Biosphere Reserve where clusters are found established at a mean height of 1.3 m [26]. Similar to other large tank bromeliads, *Ae. bracteata* individuals offer permanent shelter to a wide diversity of organisms, both specialists and opportunists [23,26,31,32], and during extreme flooding and other climatic events they constitute ecological refuges for many other ground-dwelling arthropods [33]. As with most myrmecophytes, *Ae. bracteata* can be associated with several ant species, including *N. villosa* [26]; however, it does not depend on ants for its germination [34].

*Neoponera villosa* is not an obligate inhabitant of myrmecophytes; however, in the southern region of the Yucatan Peninsula, this ant uses the tank bromeliad as a nest throughout the year, displaying a very marked local specialization [25]. There is little knowledge regarding the evolution of host–plant specialization between plants and ants in facultative associations. In the case of ants that nest in specific plants, it has been shown that host plant recognition is primarily based on the following two factors: an innate (genetically determined) attraction towards certain plants rather than others, and the influence of the environment, during development and early adult life (preimaginal learning and conditioning through contact with the host plant during larval life and the first days of adult life), that may even supplant a genetically determined attraction or deterrence [25,35,36,37]. For instance, the African arboreal ants *Tetramorium aculeatum* (Mayr) (Myrmicinae) and *Oecophylla longinoda* (Latreille) (Formicinae) present a familiarization process (early learning) that can replace the innate attraction of both species [36,37]. This learning only takes place during the neonatal stage, a sensitive period after which the influence of the environment ceases [see 35]. Attraction to *Ae. bracteata* by alate queens (gynes) and young *N. villosa* workers (nurses) has been studied in the context of new colony foundation by foundress females [25]. Gynes from colonies nesting in *Ae. bracteata* are attracted to this bromeliad, a preference that appears to be learned during the larval stage. This preimaginal learning can be further strengthened at the beginning of the imago life, causing local fidelity toward *Ae. bracteata* over other available species [25]. However, nest site selection in *N. villosa* has not been studied in the context of nest relocation, a distributed, nonhierarchical decision-making process which is performed by several scout ants who find potential nest sites. Informed scouts lead nestmates to the chosen new nest sites through tandem running, with only one nestmate being recruited at a time. The new nest site is defined by a quorum sensing mechanism, i.e., when more ants are present at one of the alternatives [5].

The Yucatan Peninsula has been identified as a region that is affected by hurricanes and droughts [38], which can result in bromeliads dislodging from their host tree and falling to the ground, thus, requiring complete ant colonies to relocate. For cavity-nesting species such as *N. villosa,* there is only a limited number of potential nest sites that can meet the requirements of a mature colony. Furthermore, nesting sites are competitively searched for by other species, specifically *Dolichoderus bispinosus* (Olivier) and *Nasutitermes* sp. [23,26]. In most cases, scouts encounter various candidate shelters and have to decide which is the most suitable. Some characteristics of the potential nest site, in particular the size of the nesting cavity, can constrain colony growth [39,40,41,42,43] and this factor is expected to influence nest site choice in *N. villosa* [23]. Furthermore, some ant species can assess nest site suitability through various physical characteristics such as darkness, cavity height, entrance width, and configuration [44,45]. However, with regard to *N. villosa*, the stimuli which intervene during nest site selection have not been identified. In various species of ants that establish obligate interactions with plants, it has been demonstrated that host plant recognition is primarily based on chemical cues that attract foundresses [46,47,48,49,50,51]. However, plant height, nest site geometry, or clear areas around trees that provide information on the size of the potential nest candidate or on its protective potential, are used by various animal species as cues during nest site selection [42,52,53,54,55] and could also play an important role during nest relocation in *N. villosa*.

In the present study, we performed different experiments (two-choice bioassays) to determine how *N. villosa* workers select a nest site in the eventuality of nest relocation. Because rearing workers from egg to adult was not feasible, we took advantage of the fact that *N. villosa* nests almost exclusively in cavities of live trees in northern Yucatan where *Ae. bracteata* is rare, to investigate nest site selection of *N. villosa* workers without previous contact with this bromeliad. Our research addressed the following questions: (1) Do *N. villosa* workers have an innate preference for *Ae. bracteata*? (2) Is the preference modulated by the preimaginal or neonatal ant experience linked to the origin of the colony (workers with or without previous contact with *Ae. bracteata*)? (3) Are the recognition and localization of *Ae. bracteata* regulated by chemical stimuli? (4) Does *Ae. bracteata* size influence nest site selection?

## 2. Materials and Methods

### 2.1. Ant Collection and Identification

Ants in bromeliads were collected in the following five sites in the southern part of the Yucatan Peninsula: Ejido Blasillo (18°7′37.98″N, 89°20′20.93″W, 261 m.a.s.l.), Nuevo Becal (18°36′39.36″ N, 89°16′15.54″ W, 239 m.a.s.l.), and Zoh-Laguna (18°35′11.61″ N, 89°25′4.67″ W, 257 m.a.s.l.) in Campeche; and Kohunlich (18°25′31.08″N, 88°48′9.89″W, 143 m.a.s.l.) and Sian Ka’an Biosphere Reserve (19°41′56.17″ N, 87°50′18.31″ W, 18 m.a.s.l.) in Quintana Roo. Ants nesting in tree cavities (mainly *Lysiloma latisiliquum* (L.) Benth., *Caesalpinia gaumeri* (Britton and Rose) Greenm., and *Leucaena leucocephala* (Lam.) de Wit (Fabaceae), and *Bursera simaruba* (L.) Sarg. (Burseraceae)) were collected essentially in Cuxtal Ecological Reserve (20°51′46.58″ N, 89°36′40.68″ W, 17 m.a.s.l.) in Yucatan, in the northern part of the Peninsula, but a few were collected in the south, in Chetumal (18°32′37.90″ N, 88°15′46.38″ W, 10 m.a.s.l.) in Quintana Roo. In the latter two sites, *Ae. bracteata* is rare or absent. Each epiphyte was cut off from the supporting branch, dismantled leaf-by-leaf in plastic bins coated with Fluon (Whitford GmbH, Diez, Germany), and all *N. villosa* ants and their brood were collected. Both ants from epiphytes and those from tree cavities were housed in plastic jars under laboratory conditions until bioassays (see below).

*Neoponera villosa* belongs to the neotropical species complex of *N. foetida* (L.), which includes 12 other species [56,57]. Due to their morphological similarity, *N. villosa* has been confused in the past with two other species with a wide distribution, *N. inversa* (Smith) and *N. curvinodis* (Forel). Until now, however, only *N. villosa* has been reported in the Yucatan Peninsula [58]. Nevertheless, in order to confirm ant identity and further support our comparisons, five workers nesting in bromeliads and five workers nesting in live trees were DNA extracted and barcoded as part of an independent study (Lachaud and Pérez-Lachaud, unpubl.). DNA extraction and amplification followed the protocol in [59], with a freezing step after initial incubation according to the recommendation of [60] for Hymenoptera. Sequences were edited using CodonCode v. 3.0.1 (CodonCode Corporation, Dedham, MA, USA) and uploaded to the Barcode of Life Database (BOLD, boldsystems.org). Voucher specimens were deposited in the Formicidae Collection of El Colegio de la Frontera Sur at Chetumal, Quintana Roo, Mexico (ECO-CH-F).

### 2.2. Nest Site Selection

As our study is focused on nest relocation, only workers were used for the two-choice tests implemented to evaluate nest site selection. This parallels nest relocation following disturbance or destruction of the old nest, whereby emigrations are organized by workers (scouts) that set out from the damaged nest to find a new home, thoroughly inspecting any candidate nest that they find [2]. As in various other ponerine species such as *N. verenae* (Forel) (referred to as *Pachycondyla obscuricornis* Emery [61]), *N. apicalis* (Latreille) [62], or *Diacamma indicum* (Santschi) [63,64], *N. villosa* uses a specific behavior called tandem running in which a single worker attracts a single recruit (or two in some occasions) and leads her towards the new nest site [17]. As in other *Neoponera* [61,62], such recruitment by *N. villosa* is exclusively used during nest relocation and never during foraging, see [18].

#### 2.2.1. Experimental Setup

Ants from 35 colonies living in *Ae. bracteata* and 11 colonies (or parts of colonies) nesting in tree cavities were used in bioassays (Table S1). Observations were conducted from 26 January 2017 to 12 February 2018. Bioassays were carried out two weeks after field collection; during this period of acclimatization, and during bioassays, ants were held under natural illumination and at room temperature (26 ± 1 °C). Workers were randomly selected from the original nest and only evaluated once. Two-choice tests were conducted using transparent plastic jars (14 cm in diameter × 25 cm in height, 3 L vol.) into which the ants were deposited. Each jar was connected via a transparent plastic tube (1.5 cm in diameter × 20 cm in length) to an election chamber (bioassays with live plants: 45 × 30 × 60 cm plastic box; bioassays with parts of plants (leaves): 40 × 21 × 14 cm). Each election chamber included a glass tube (2 cm in diameter x 8 cm in length) filled with water and stuffed with cotton at one end. The ants were fed sliced apple pulp, which was placed in the election chamber for the duration of the bioassay.

The protocol for the observations followed that of [25]. Behavioral heterogeneity among colony members is common in insect societies and individual behavioral specialization during nest moving has been signaled, for example, in the model ant genus *Temnothorax* [65,66]; however, specialized *Temnothorax* workers are readily replaced in removal experiments showing organizational resilience of ant colonies [65]. As colony size in *N. villosa* varies greatly from one colony to another, and because it was not feasible to collect a sufficient number of complete colonies to perform the required number of replicates per bioassay, a fixed sample size of 20 ants per bioassay was used. Furthermore, this is a common procedure in experiments with large ants (see for example [36,37]). For each bioassay, two different refuges or “nest sites”, consisting of tubular shelters to eliminate the influence of plant architecture, were placed in the election chamber. Subsequently, a group of 20 workers randomly obtained from those foraging and some brood were gently placed into the adjacent transparent jar. We carried out 21 to 30 replicates for each comparison and each replicate consisted of individuals from the same colony. Then, the experimental device was closed and set aside for 24 h, allowing the ants to install themselves in one shelter along with the brood (see [36]). The stimulus for the initiation of movement towards a potential nest (no shelter in the jar and artificial illumination) was constant across experiments and across replicates within experiments. This is a standard procedure used to trigger colony relocation in ants [67]. After 24 hours we evaluated the number of workers in any of the two refuge options (“nest sites”) and those that remained in the jar or that were wandering or foraging. 

#### 2.2.2. Experiment One

To evaluate whether *N. villosa* nests in any available cavity or whether it prefers the refuge provided by *Ae. bracteata*, workers were presented with two tubular shelters (4 cm in diameter × 10 cm in length, with only one opening) made from the following: (a) the rolled leaves of *Ae. bracteata* (treatment) and (b) from a cardboard (control). Thirty replicates were performed with workers originating from colonies living in bromeliads and thirty with workers from colonies collected in tree cavities.

#### 2.2.3. Experiment Two

To evaluate whether *N. villosa* workers are able to discriminate *Ae. bracteata* through chemical stimuli emitted by the plant, shelters made of leaves of two other species belonging also to the Bromeliaceae family (*Ae. bromeliifolia* (Rudge) Baker and *Ananas comosus* (L.) Merr.) where offered in combination with *Ae. bracteata* in two-choice bioassays, as in the previous experiment. Thirty replicates were performed for each comparison with workers originating from colonies living in bromeliads, and 21 to 26 replicates with ants from colonies collected in tree cavities. For each replicate, the ants had the choice between two shelters, i.e., one shelter made from the leaves of *Ae. bracteata* (control), and another made from the leaves of one of the two other bromeliad species. 

#### 2.2.4. Experiment Three

To evaluate the influence of other bromeliad-related traits (structure of the plant), ants were offered the choice between whole plants of similar size of the two *Aechmea* species. Thirty replicates were performed with both types of workers.

#### 2.2.5. Experiment Four

As the results of the previous experiments showed that both shelters made of *Ae. bracteata* leaves and whole plants of this species were preferred (see Results), we evaluated whether the choice by *N. villosa* ants could be influenced by the size of the available *Ae. bracteata*. Twenty-nine replicates with workers from colonies nesting in *Ae. bracteata* were set up. In each replicate, *Ae. bracteata* bromeliads of two different sizes were offered, i.e., small (25 cm) vs. large (80 cm).

### 2.3. Statistical Analyses

Not all ants were found inside refuges; some workers were foraging, and others were inactive. Inactivity is very common in social insects and is an intrinsic feature of the ants’ behavior [68], making up to 40% of the members in a colony (e.g., [69]); furthermore, specific workers are consistently inactive [70]. Inactive workers are quantitatively important in *N. villosa* colonies [19]. To avoid inconsistencies due to a number of ants not choosing or performing other activities, we calculated the total number of ants found inside the proposed refuges or plants and used proportions of ants as the variable response.

Data (successes and failures) were analyzed fitting a generalized linear mixed model (GLMM) with a binomial error distribution and a logit link (maximum likelihood). To control for any bias due to colony-level effects, colony was included as a random effect, and the treatments (with or without experience with *Ae. bracteata*) as a fixed effect. To explore the magnitude of the fixed effect, we performed a likelihood ratio test (Wald Chi-square test); 95% CI allowed to infer differences within treatments. Analyses were performed in package LME4 in R version 3.6.2 [71,72].

### 2.4. Ethics Statement

Sampling complied with the current laws of Mexico and was carried out under permit number FAUT-0277 from Secretaría de Medio Ambiente y Recursos Naturales, Dirección General de Vida Silvestre, granted to G.P.-L. Only the biological material required for this study was collected.

## 3. Results

### 3.1. Species Identification

DNA sequences generated in the present work (Genbank Accession Numbers MK779595 to MK779604) confirmed that both populations (ants nesting in *Ae. bracteata* and ants nesting in live tree cavities) did not diverge genetically. All ants used in this work belonged to *N. villosa*. DNA sequences of both populations represent a single molecular operational taxonomic unit, and cluster with all other *N. villosa* molecular public data (Figure S1).

### 3.2. Tandem Running Behavior

In the bioassays, *N. villosa* workers began exploring both the new area (election chamber) and the proposed refuges, and then selected one shelter. Afterwards, workers returned to the nest box and recruited nestmates through tandem running behavior. These recruited workers moved to the selected refuge, inspected it, and returned to the “old nest” to recruit new nestmates. Qualitatively, we found that several tandem running ants followed the same path, suggesting trail laying behavior, although marking of the trail was not observed (Video S1). Similar trail laying through hind gut fluids or pygidial gland secretions has been reported in other ponerine species using tandem running [73,74,75,76]. The recruitment process was initiated through a "jerking" movement of a recruiting ant stimulating a nestmate to follow her to the new nest site, whereby the ant performs a rapid and vertical shaking of the body. Such rapid, vertical shaking of the body displayed by the recruiting ant to enhance the chemical signal has been reported in other ant species from various subfamilies [77,78]. Nestmates reacted by replicating the jerking movements, and then initiated tandem running along the trail of the recruiting ant toward the new nest site. A single worker, or occasionally a maximum of two, were recruited and travelled in a single column. In some cases, when contact between the scout and the follower was broken, the recruiting ant pulled the legs of the nestmate with their jaws to reinforce the recruitment signal.

### 3.3. Nest Site Selection

#### 3.3.1. Experiment One

**Figure 1:**
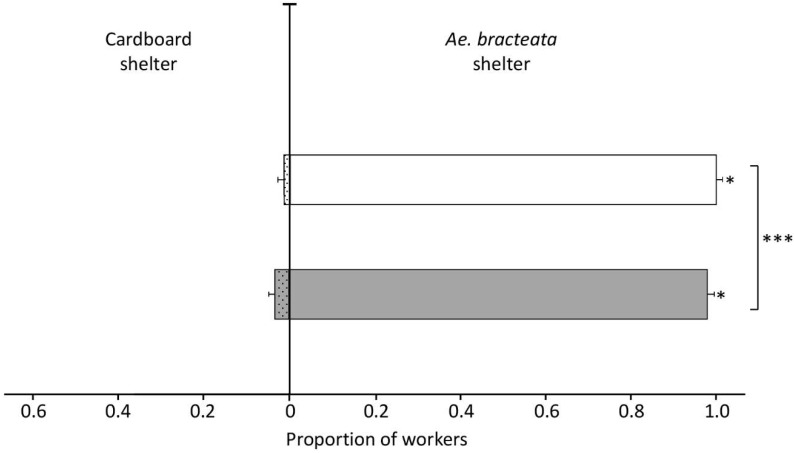
Mean proportion of *N. villosa* workers (± SEM) in shelters consisting of *Ae. bracteata* leaves (empty bars) vs. cardboard shelters (dotted bars). Workers from colonies of two distinct origins were tested, i.e., ants originally nesting in *Ae. bracteata* (n = 30 trials, grey bars) and ants from colonies previously nesting in tree cavities (n = 30 trials, white bars). * *p* ˂ 0.05, *** *p* ˂ 0.0001, GLMM Wald χ^2^.

Ants from both origins (whether originally nesting in bromeliads or in tree cavities) significantly preferred refuges made up of the leaves of *Ae. bracteata* over cardboard shelters: 0.978 ± 0.0002 (mean proportion ± SEM, 95% CI [0.9774–0.9781]) for ants originally nesting in *Ae. bracteata* and 0.989 ± 0.0001 (95% CI [0.9892–0.9897]) for those nesting in tree cavities (Figure 1). However, the origin of ants had a significant influence on their choices (GLMM, Wald χ^2^ = 8338.5, df = 1, *p* ˂ 0.0001) as ants originally nesting in bromeliads had a significantly lower probability of choosing the refuge made up of leaves of the bromeliad than ants originally nesting in tree cavities. In general, the brood was transported to the shelters with the higher proportion of workers.

#### 3.3.2. Experiment Two

**Figure 2:**
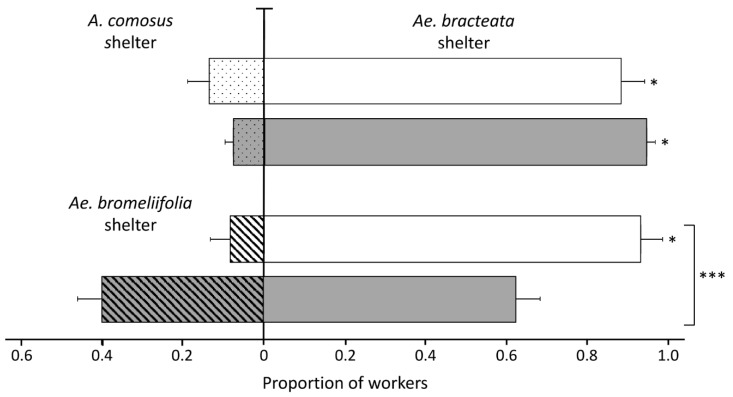
Mean proportion of *N. villosa* workers (± SEM) in shelters consisting of leaves of *Aechmea bracteata* (empty bars) vs. leaves of two other bromeliads, *Ae. bromeliifolia* (stripped bars) or *Ananas comosus* (dotted bars). Workers from colonies of two distinct origins were tested, i.e., workers originally nesting in *Ae. bracteata* (n = 30 trials, grey bars) and workers from colonies previously nesting in tree cavities (n = 21 or n = 26 trials, white bars). * *p* ˂ 0.05, *** *p* ˂ 0.0001, GLMM Wald χ^2^.

When ants had to select between refuges made up of leaves of two *Aechmea* species, their choice was significantly influenced by their previous nesting site (GLMM, Wald χ^2^ = 13.067, *p* = 0.00035). Ants from tree cavities that did not have any previous contact with *Ae. bracteata* have a higher probability of choosing *Ae. bracteata* over *Ae. bromeliifolia* (0.960 ± 0.027, 95% CI [0.8592–0.9895]) than ants from colonies originally nesting in *Ae. bracteata* (0.581 ± 0.089, 95% CI [0.4043–0.7391]). Ants from colonies nesting in *Ae. bracteata* did not show any marked preference (Figure 2). However, when the choice concerned refuges made up of leaves of species from two different Bromeliaceae genera, ants from both origins preferred *Ae. bracteata* over *A. comosus* (ants nesting in bromeliads: 0.952 ± 0.018, 95% CI [0.9015–0.9776]; ants in tree cavities: 0.929 ± 0.033, 95% CI [0.8296–0.9720]) (Figure 2); and although this pattern was stronger in ants originally nesting in *Ae. bracteata*, the probability of choosing between *Ae. bracteata* and *A. comosus* was not influenced by the origin of ants (GLMM, Wald χ^2^ = 0.486, *p* = 0.48).

#### 3.3.3. Experiment Three

**Figure 3:**
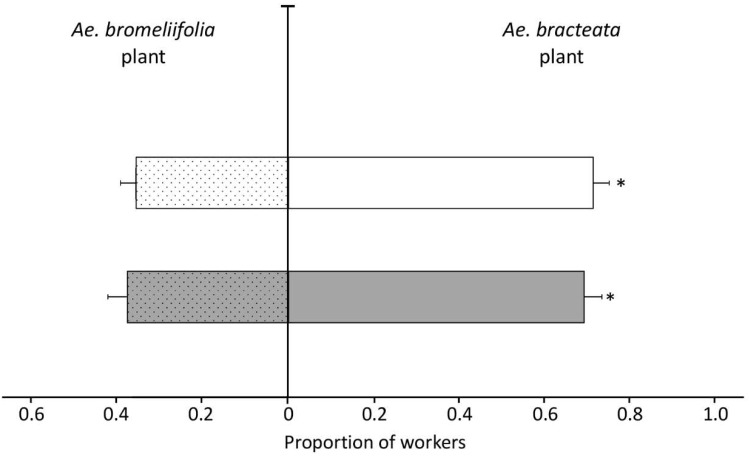
Mean proportion of *N. villosa* workers (± SEM) in bioassays of whole plants of *Ae. bracteata* (empty bars) vs. *Ae. bromeliifolia* (dotted bars) of the same size. Bioassays were performed with two different experimental groups, i.e., workers originating from colonies originally nesting in *Ae. bracteata* (n = 30 trials, grey bars) and workers from colonies collected in tree cavities (n = 30 trials, white bars). * *p* ˂ 0.05, GLMM Wald χ^2^.

The origin of the ants had no effect on the probability of choosing between whole plants of *Ae. bracteata* and *Ae. bromeliifolia* (GLMM, Wald χ^2^ = 0.011, *p* = 0.916). Ants of both origins preferred *Ae. bracteata* over *Ae. bromeliifolia* plants (ants originally nesting in *Ae. bracteata*: 0.661 ± 0.034, 95% CI [0.5915–0.7249]; ants nesting in tree cavities: 0.667 ± 0.041, 95% CI [0.5814–0.7430]) (Figure 3).

#### 3.3.4. Experiment Four

**Figure 4:**
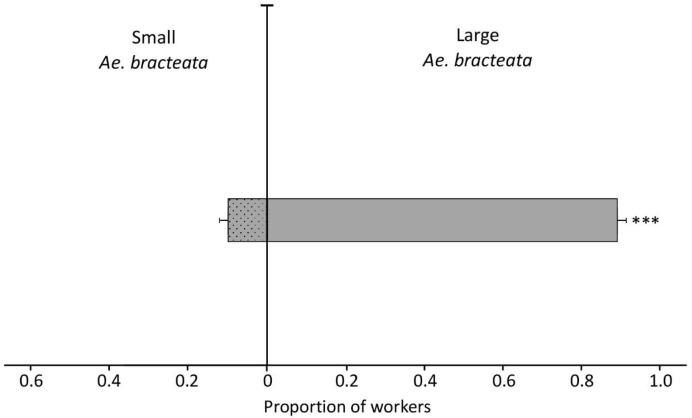
Mean proportion of *N. villosa* workers (± SEM) in bioassays with two *Ae. bracteata* plants of different sizes, large (80 cm) vs. small (25 cm). All workers tested were originally nesting in *Ae. bracteata* (n = 29 trials). *** *p* < 0.0001, GLMM.

*Neoponera villosa* workers originating from colonies established in *Ae. bracteata* significantly chose large *Ae. bracteata* bromeliads over small ones (GLMM, Wald type Z = 10.51, df = 1, *p* ˂ 0.0001; Figure 4). The mean proportion of ants choosing the large over the small bromeliad was 0.899 ± 0.025, 95% CI [0.8489–0.9510].

## 4. Discussion

Most animals, if not all, exhibit innate behaviors in response to specific sensory stimuli [79,80]. Bumble bees and honeybees, for example, exhibit innate color preferences, notably yellow and purple, which reflect the peak sensitivity of their color receptors [81,82,83,84], while the pseudomyrmecine ants *Tetraponera aethiops* Smith show an innate attraction to their natural host plant *Barteria fistulosa* Mast. (Passifloraceae), even when young callows were reared in laboratory conditions without any further contact with this plant post emergence [37]. Our experiments provide a similar example of innate attraction of *N. villosa* workers towards the bromeliad *Ae. bracteata*. Whatever the origin of the ants (with or without previous experience with *Ae. bracteata*), our data show that *N. villosa* workers exhibit a significant preference for refuges made up of leaves of *Ae. bracteata* over other available potential refuges consisting of leaves of another bromeliad species, *A. comosus*, or of cardboard; however, ants with a previous experience with *Ae. bracteata* did not differentiate between refuges made up of leaves of this bromeliad and those of the close *Ae. bromeliifolia*. Contrary to the results obtained by [25], which did not demonstrate any spontaneous preference of *N. villosa* for *Ae. bracteata* (workers reared in the laboratory without any contact with plants were attracted indifferently towards *Ae. bracteata* or towards the orchid *Myrmecophila tibicinis* (Batem.) Rolfe), our results point to the existence of an innate preference for *Ae. bracteata*. In all of the bioassays, workers without previous experience with this bromeliad significantly preferred *Ae. bracteata*, although a proportion of ants did not engage in emigrations (inactive ants). 

The influence of the environment, through preimaginal and neonatal learning (early experience), can interfere and replace any innate attraction or repulsion [85,86]. In ants, environmental induction of adult choices by passive familiarization during early adulthood has been demonstrated for various species. For example, although under natural conditions thyme (*Thymus vulgaris* L.) repels adult workers of the formicines *Formica polyctena* Foerster and *Camponotus vagus* Scopoli, workers of these species chose to settle in tubes that contained this plant if they have been reared in its presence when they were callow neonates [35,87]. Similarly, in the African arboreal ants *T. aculeatum* and *O. longinoda*, early learning during the first part of the life of adult workers and gynes can supersede an innate attraction to guava (*Psidium guajava* L.) and cocoa (*Theobroma cacao* L.) or to mango leaves (*Mangifera indica* L.), respectively [36]. Early learning, during the larval life and/or just after adult emergence, appears to occur to some extent in *N. villosa*, as our data show that the preference for *Ae. bracteata* was modulated by the original nesting substrate; previous experience with *Ae. bracteata* enhancing the preference towards this plant over cardboard or *A. comosus*. These results confirm previous studies on foundresses of *N. villosa* which have shown that the influence of the original nest site environment on subsequent individual choice during nest site selection for colony foundation is due to imprinting during larval life, strengthened at the beginning of the imago life through early learning [25]. When given the choice between a refuge containing *Ae. bracteata* or nothing, gynes of *N. villosa* that have previously experienced contact with the bromeliad during their larval life and the first part of their pupal stage significantly preferred *Ae. bracteata*, whereas gynes which completed the pupal stage on another epiphyte (*M. tibicinis*) did not discriminate between both options. However, gynes which completed their pupal life on *M. tibicinis* displayed a significant preference to this plant, when presented with a choice between *Ae. bracteata* and *M. tibicinis* [25]. The local fidelity towards *Ae. bracteata* over other available nest sites observed in our study area and the marked preference for this epiphyte during our experiments could be explained through early learning by winged queens, as suggested by [25], combined with an innate attraction to *Ae. bracteata* in workers, influencing their choice during nest relocation events. The evolution of such an innate attraction, leading to local specialization in *Ae. bracteata* as a nesting site, could be due to the predominance of this particular bromeliad throughout the biogeographic area of *N. villosa* (*Ae. bracteata* occurs from E Mexico to N Colombia and NW Venezuela [88]), and also because this bromeliad as a microhabitat provides specific benefits to ants, both as a shelter and as a foraging site, and further constitutes a refuge during extreme stochastic climatic events [33,89]. Preference of *N. villosa* workers for *Ae. bracteata* could be an adaptive response driven by climatic events (droughts, floods) in our study area, promoting the selection of such a stable microhabitat.

Various other bromeliad associated traits (size, chemical profile) could also be involved in nest site selection as demonstrated by the preference of *N. villosa* workers for large *Ae. bracteata* when presented with small and large plants, or by the differences in preference demonstrated when choosing between closely related bromeliad species (both when offered as refuges and complete plants), most likely related to the chemical substances they produce. The bromeliads used in this study share similar traits (e.g., long and narrow leaves), and the texture of their leaves and general architecture are similar, but not identical; furthermore, the nature and composition of their chemical signals are different, particularly between species from different genera [90], and it is possible that ants use any small difference in structure or composition of the leaves during nest site selection. In addition, the architectural form and complexity of the plant could facilitate the emergence of different temperature and humidity microhabitats suitable for ants to settle, and it is known that other specialist arthropods (e.g., bromeliad-dwelling salticids) choose bromeliads based on rosette and leaf architectures [91]; furthermore, larger individual plants with a complex structure facilitate the development of large ant populations and promote the maintenance of a diverse community of potential prey [92]. The use of chemical cues for host localization has been reported in a wide range of insects, including both herbivores [93,94,95,96] and predators or parasites [97,98,99]. In ants, chemical volatiles are also used to identify potential host plants. For example, *Crematogaster* spp. foundress queens can recognize their *Macaranga* host plant species, identifying chemical compounds of the stem surfaces of seedlings [46]; and queens of *Azteca* spp. and *Allomerus octoarticulatus* Mayr use chemical cues to select their myrmecophyte *Cordia nodosa* Lam. [47]. In our experiments, it was evident that *N. villosa* workers, originally nesting in *Ae. bracteata*, had difficulty in arriving at a consensus when in the presence of shelters of two *Aechmea* species, without further information on the suitability of the potential nest sites (plant structure or size of the cavity, for example), and therefore the probabilities of choosing either bromeliad were similar (Figure 2). Contrastingly, choosing between complete plants of these two bromeliads was straight forward and significantly in favor of *Ae. bracteata* over the other species, although some ants also settled inside *Ae*. *bromeliifolia* plants. Noteworthy, a proportion of ants did not engage in emigrations (inactive ants). Our failure to show any statistical difference when ants chose between refuges of these two bromeliads could have arisen, in part, due to the presence of inactive workers and the possible exclusion of specialized workers in our reduced experimental groups. This could have influenced the number of ants in shelters after 24 h, as a significant higher latency in performing emigration tasks has been reported for workers not specialized on emigration tasks in *Temnothorax* ants [65]. Considered together, these results suggest that workers of *N. villosa* select *Ae. bracteata* through some plant stimuli, probably of chemical nature, supplemented with information provided by the whole plant. The ability to discriminate between distinct plant species and genera is an obvious advantage, as the time and energy to find a suitable nest site is minimized.

Evidently, nearly all ant species have the capability of shifting their nests if they become unsuitable [1] and selecting the best nest site among numerous alternatives can be critical to the success and survival of the colony. As our results show, in most instances, *N. villosa* workers preferred to settle in *Ae. bracteata* shelters and plants over other possibilities, and preferred large *Aechmea* plants over small ones. Behavioral flexibility constitutes an essential component of the adaptive repertoires of animals. In this context, it is not impossible that modulation of the innate attraction of *N. villosa* workers towards *Ae. bracteata* through early experience could facilitate the replacement of this plant as the most suitable nest site in habitats where *Ae. bracteata* is rare, as occurs in the northern part of the Yucatan Peninsula where this species nests in cavities of several live trees.

## 5. Conclusions

In our experiments, nest site choice by *N. villosa* workers was influenced by an innate attraction to the bromeliad *Ae. bracteata*. The local fidelity towards *Ae. bracteata* over other available nest sites in the southern region of the Yucatan Peninsula and the marked preference for this epiphyte in our experiments could be explained through this innate attraction to *Ae. bracteata* influencing the choice of workers during nest relocation events. Reinforcement of this preference by preimaginal and early learning during adulthood is not excluded, but more experiments are needed specifically targeting early learning in this species. Preference of *N. villosa* workers for *Ae. bracteata* could be an adaptive response driven by extreme climatic events in our study area, promoting the selection of such a stable microhabitat as a nesting site.

## Supplementary Materials

The following are available online at https://www.mdpi.com/2075-4450/11/3/200/s1, Table S1: Original composition of the *Neoponera villosa* colonies used in the two-choice bioassays, Figure S1: Taxon ID tree of *N. villosa* molecular public data, including the 10 sequences from this study (highlighted in yellow), Video S1: Characteristic recruitment behavior in *Neoponera villosa*. Note that two tandem pairs are following the same path, suggesting the existence of chemical trail laying.
