# Supplementary material for: Nest Site Selection during Colony Relocation in Yucatan Peninsula Populations of the Ponerine Ants Neoponera villosa (Hymenoptera: Formicidae)
Source: Insects. 2020 Mar 23;11(3):200. doi: 10.3390/insects11030200 (PMC7143209; doi:10.3390/insects11030200)
Supplement: Supplementary file 1 [file insects-11-00200-s001.zip › insects-708056-supple/Rocha et al -Supp Material.pdf]

Electronic Supplementary Material –

Nest-site selection during colony relocation in Yucatan Peninsula populations of the ponerine ant *Neoponera villosa* (Hymenoptera: Formicidae)

Franklin H. Rocha[1], Jean-Paul Lachaud[1,2], Yann Hénaut[1], Carmen Pozo[1] & Gabriela Pérez-Lachaud[1*]

[1] El Colegio de la Frontera Sur, Conservación de la Biodiversidad, Avenida Centenario km 5.5, Chetumal 77014, Quintana Roo, México. [2] Centre de Recherches sur la Cognition Animale (CRCA), Centre de Biologie Intégrative (CBI), Université de Toulouse; CNRS, UPS, Toulouse, France

*Corresponding author (igperez@ecosur.mx)

CONTENT

Table S1. Original composition of the *Neoponera villosa* colonies used in the two-choice bioassays.

Fig. S1. Taxon ID tree of *N. villosa* molecular public data, including the 10 sequences from this study (highlighted in yellow).

Video S1. Characteristic recruitment behavior in *Neoponera villosa*. Note that two tandem pairs are following the same path, suggesting the existence of chemical trail laying.

Table S1. Composition of the *Neoponera villosa* colonies used in the two-choice bioassays. Data for colonies nesting in tree cavities correspond to complete colonies collected. Since ants nested in live trees most colonies were incomplete.

Colonies nesting in *Aechmea bracteata*					
Type of colony	n	Mean number of queens	Mean number of workers	Mean number of larvae	Mean number of cocoons
Monogynic	10	1	84.5 ± 16.4	33.7 ± 6.1	44.5 ± 10.3
Polygynic	21	5.4 ± 0.7	128 ±17.4	54.2 ± 12.6	54.8 ± 12.0
Queenless	4	-	50 ± 5.0	20.5 ± 12.9	20.3 ± 8.0
Colonies nesting in tree cavities					
Monogynic	5	1	117.3 ± 64.1	14.0 ± 10.1	19.7 ± 8.4

Fig. S1. Taxon ID tree of N. villosa molecular public data, including the 10 sequences from this study (highlighted in yellow).

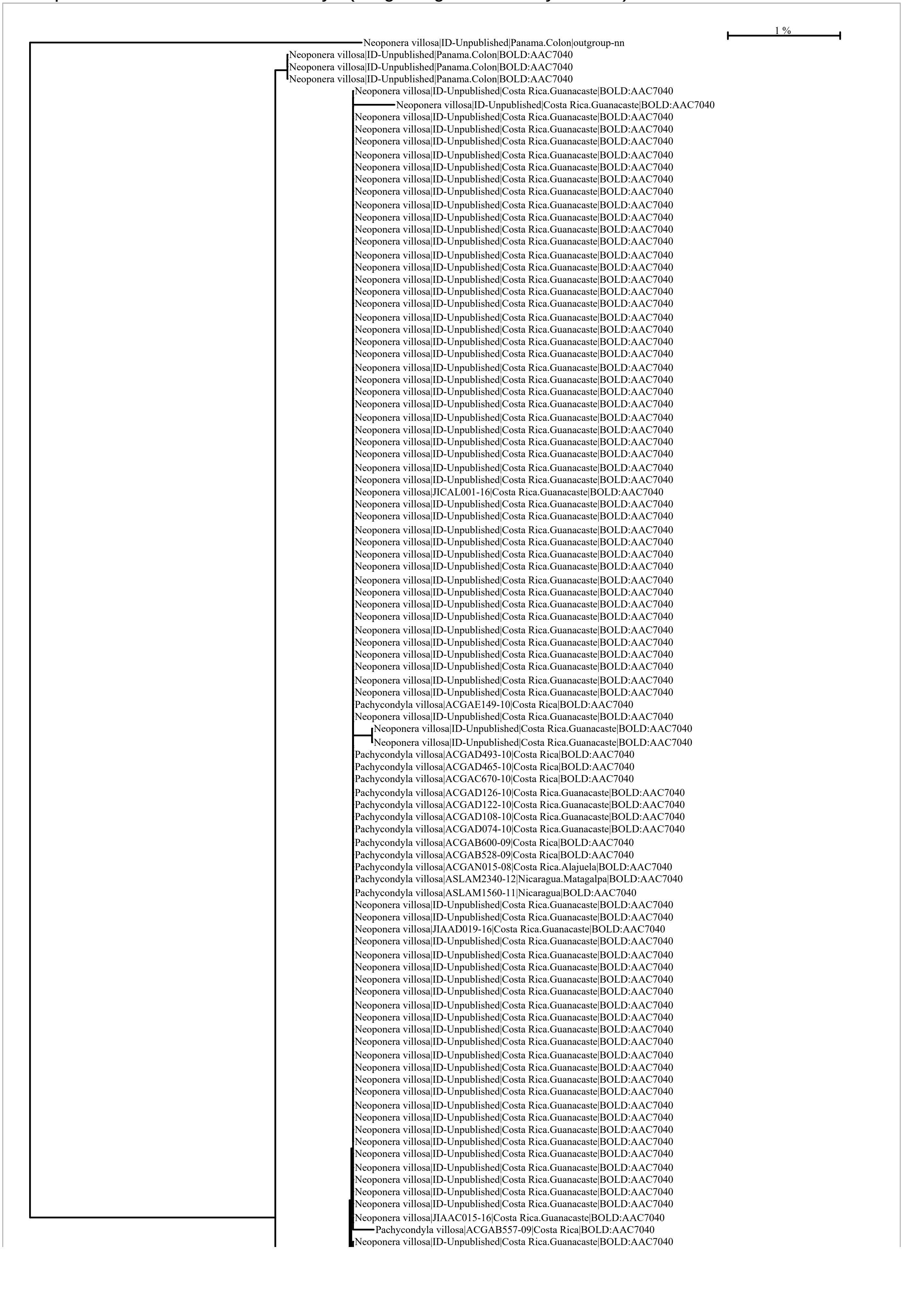

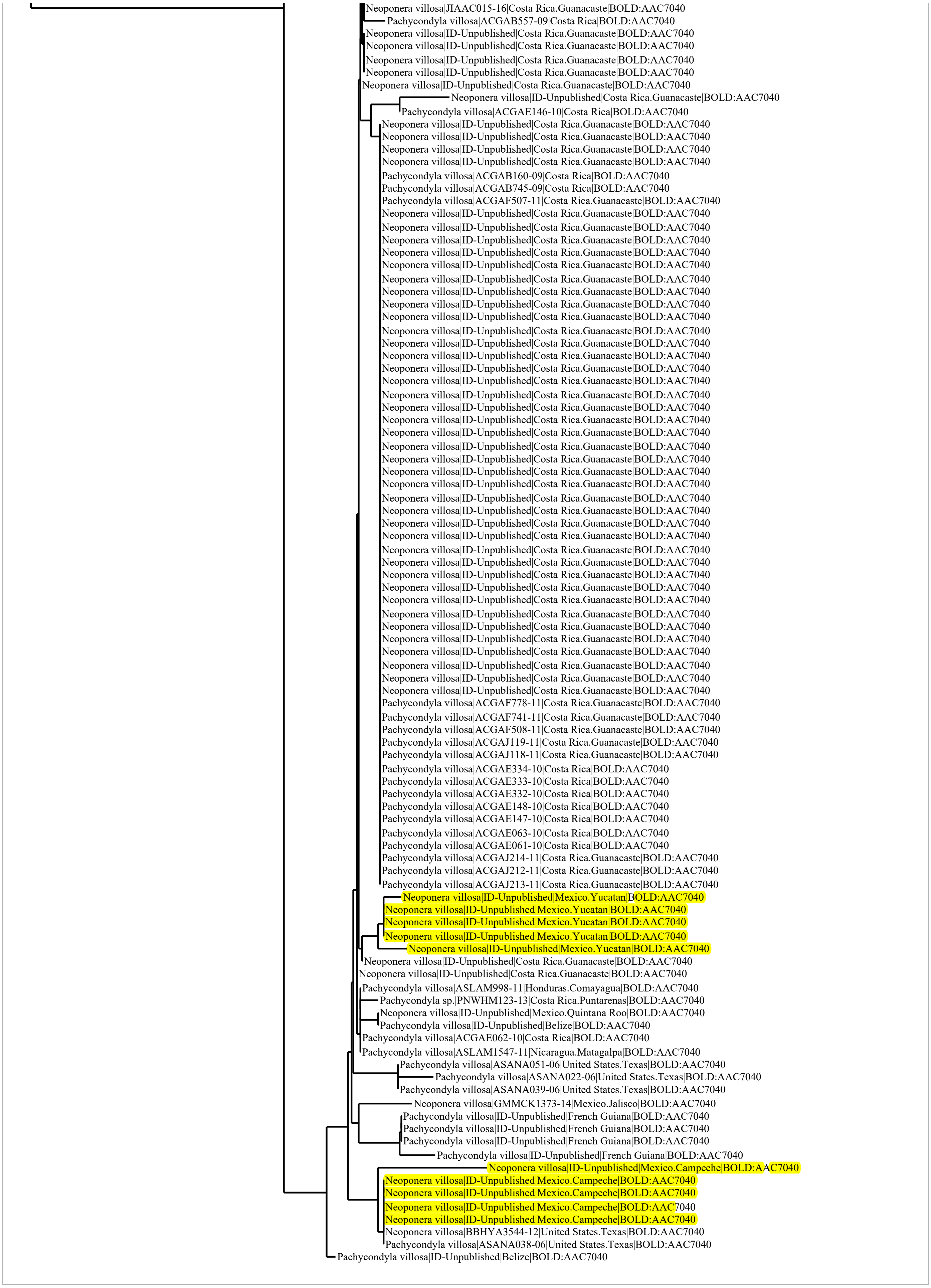

Neoponera villosa|JIAAC015-16|Costa Rica.Guanacaste|BOLD:AAC7040
Pachycondyla villosa|ACGAB557-09|Costa Rica|BOLD:AAC7040
Neoponera villosa|ID-Unpublished|Costa Rica.Guanacaste|BOLD:AAC7040
Neoponera villosa|ID-Unpublished|Costa Rica.Guanacaste|BOLD:AAC7040
Neoponera villosa|ID-Unpublished|Costa Rica.Guanacaste|BOLD:AAC7040
Neoponera villosa|ID-Unpublished|Costa Rica.Guanacaste|BOLD:AAC7040
Neoponera villosa|ID-Unpublished|Costa Rica.Guanacaste|BOLD:AAC7040
Neoponera villosa|ID-Unpublished|Costa Rica.Guanacaste|BOLD:AAC7040
Pachycondyla villosa|ACGAE146-10|Costa Rica|BOLD:AAC7040
Neoponera villosa|ID-Unpublished|Costa Rica.Guanacaste|BOLD:AAC7040
Neoponera villosa|ID-Unpublished|Costa Rica.Guanacaste|BOLD:AAC7040
Neoponera villosa|ID-Unpublished|Costa Rica.Guanacaste|BOLD:AAC7040
Neoponera villosa|ID-Unpublished|Costa Rica.Guanacaste|BOLD:AAC7040
Pachycondyla villosa|ACGAB160-09|Costa Rica|BOLD:AAC7040
Pachycondyla villosa|ACGAB745-09|Costa Rica|BOLD:AAC7040
Pachycondyla villosa|ACGAF507-11|Costa Rica.Guanacaste|BOLD:AAC7040
Neoponera villosa|ID-Unpublished|Costa Rica.Guanacaste|BOLD:AAC7040
Neoponera villosa|ID-Unpublished|Costa Rica.Guanacaste|BOLD:AAC7040
Neoponera villosa|ID-Unpublished|Costa Rica.Guanacaste|BOLD:AAC7040
Neoponera villosa|ID-Unpublished|Costa Rica.Guanacaste|BOLD:AAC7040
Neoponera villosa|ID-Unpublished|Costa Rica.Guanacaste|BOLD:AAC7040
Neoponera villosa|ID-Unpublished|Costa Rica.Guanacaste|BOLD:AAC7040
Neoponera villosa|ID-Unpublished|Costa Rica.Guanacaste|BOLD:AAC7040
Neoponera villosa|ID-Unpublished|Costa Rica.Guanacaste|BOLD:AAC7040
Neoponera villosa|ID-Unpublished|Costa Rica.Guanacaste|BOLD:AAC7040
Neoponera villosa|ID-Unpublished|Costa Rica.Guanacaste|BOLD:AAC7040
Neoponera villosa|ID-Unpublished|Costa Rica.Guanacaste|BOLD:AAC7040
Neoponera villosa|ID-Unpublished|Costa Rica.Guanacaste|BOLD:AAC7040
Neoponera villosa|ID-Unpublished|Costa Rica.Guanacaste|BOLD:AAC7040
Neoponera villosa|ID-Unpublished|Costa Rica.Guanacaste|BOLD:AAC7040
Neoponera villosa|ID-Unpublished|Costa Rica.Guanacaste|BOLD:AAC7040
Neoponera villosa|ID-Unpublished|Costa Rica.Guanacaste|BOLD:AAC7040
Neoponera villosa|ID-Unpublished|Costa Rica.Guanacaste|BOLD:AAC7040
Neoponera villosa|ID-Unpublished|Costa Rica.Guanacaste|BOLD:AAC7040
Neoponera villosa|ID-Unpublished|Costa Rica.Guanacaste|BOLD:AAC7040
Neoponera villosa|ID-Unpublished|Costa Rica.Guanacaste|BOLD:AAC7040
Neoponera villosa|ID-Unpublished|Costa Rica.Guanacaste|BOLD:AAC7040
Neoponera villosa|ID-Unpublished|Costa Rica.Guanacaste|BOLD:AAC7040
Neoponera villosa|ID-Unpublished|Costa Rica.Guanacaste|BOLD:AAC7040
Neoponera villosa|ID-Unpublished|Costa Rica.Guanacaste|BOLD:AAC7040
Neoponera villosa|ID-Unpublished|Costa Rica.Guanacaste|BOLD:AAC7040
Neoponera villosa|ID-Unpublished|Costa Rica.Guanacaste|BOLD:AAC7040
Neoponera villosa|ID-Unpublished|Costa Rica.Guanacaste|BOLD:AAC7040
Neoponera villosa|ID-Unpublished|Costa Rica.Guanacaste|BOLD:AAC7040
Neoponera villosa|ID-Unpublished|Costa Rica.Guanacaste|BOLD:AAC7040
Neoponera villosa|ID-Unpublished|Costa Rica.Guanacaste|BOLD:AAC7040
Neoponera villosa|ID-Unpublished|Costa Rica.Guanacaste|BOLD:AAC7040
Neoponera villosa|ID-Unpublished|Costa Rica.Guanacaste|BOLD:AAC7040
Neoponera villosa|ID-Unpublished|Costa Rica.Guanacaste|BOLD:AAC7040
Neoponera villosa|ID-Unpublished|Costa Rica.Guanacaste|BOLD:AAC7040
Neoponera villosa|ID-Unpublished|Costa Rica.Guanacaste|BOLD:AAC7040
Neoponera villosa|ID-Unpublished|Costa Rica.Guanacaste|BOLD:AAC7040
Neoponera villosa|ID-Unpublished|Costa Rica.Guanacaste|BOLD:AAC7040
Neoponera villosa|ID-Unpublished|Costa Rica.Guanacaste|BOLD:AAC7040
Neoponera villosa|ID-Unpublished|Costa Rica.Guanacaste|BOLD:AAC7040
Pachycondyla villosa|ACGAF778-11|Costa Rica.Guanacaste|BOLD:AAC7040
Pachycondyla villosa|ACGAF741-11|Costa Rica.Guanacaste|BOLD:AAC7040
Pachycondyla villosa|ACGAF508-11|Costa Rica.Guanacaste|BOLD:AAC7040
Pachycondyla villosa|ACGAJ119-11|Costa Rica.Guanacaste|BOLD:AAC7040
Pachycondyla villosa|ACGAJ118-11|Costa Rica.Guanacaste|BOLD:AAC7040
Pachycondyla villosa|ACGAE334-10|Costa Rica|BOLD:AAC7040
Pachycondyla villosa|ACGAE333-10|Costa Rica|BOLD:AAC7040
Pachycondyla villosa|ACGAE332-10|Costa Rica|BOLD:AAC7040
Pachycondyla villosa|ACGAE148-10|Costa Rica|BOLD:AAC7040
Pachycondyla villosa|ACGAE147-10|Costa Rica|BOLD:AAC7040
Pachycondyla villosa|ACGAE063-10|Costa Rica|BOLD:AAC7040
Pachycondyla villosa|ACGAE061-10|Costa Rica|BOLD:AAC7040
Pachycondyla villosa|ACGAJ214-11|Costa Rica.Guanacaste|BOLD:AAC7040
Pachycondyla villosa|ACGAJ212-11|Costa Rica.Guanacaste|BOLD:AAC7040
Pachycondyla villosa|ACGAJ213-11|Costa Rica.Guanacaste|BOLD:AAC7040
Neoponera villosa|ID-Unpublished|Mexico.Yucatan|BOLD:AAC7040
Neoponera villosa|ID-Unpublished|Mexico.Yucatan|BOLD:AAC7040
Neoponera villosa|ID-Unpublished|Mexico.Yucatan|BOLD:AAC7040
Neoponera villosa|ID-Unpublished|Mexico.Yucatan|BOLD:AAC7040
Neoponera villosa|ID-Unpublished|Mexico.Yucatan|BOLD:AAC7040
Neoponera villosa|ID-Unpublished|Costa Rica.Guanacaste|BOLD:AAC7040
Neoponera villosa|ID-Unpublished|Costa Rica.Guanacaste|BOLD:AAC7040
Pachycondyla villosa|ASLAM998-11|Honduras.Comayagua|BOLD:AAC7040
Pachycondyla sp.|PNWHM123-13|Costa Rica.Puntarenas|BOLD:AAC7040
Neoponera villosa|ID-Unpublished|Mexico.Quintana Roo|BOLD:AAC7040
Pachycondyla villosa|ID-Unpublished|Belize|BOLD:AAC7040
Pachycondyla villosa|ACGAE062-10|Costa Rica|BOLD:AAC7040
Pachycondyla villosa|ASLAM1547-11|Nicaragua.Matagalpa|BOLD:AAC7040
Pachycondyla villosa|ASANA051-06|United States.Texas|BOLD:AAC7040
Pachycondyla villosa|ASANA022-06|United States.Texas|BOLD:AAC7040
Pachycondyla villosa|ASANA039-06|United States.Texas|BOLD:AAC7040
Neoponera villosa|GMMCK1373-14|Mexico.Jalisco|BOLD:AAC7040
Pachycondyla villosa|ID-Unpublished|French Guiana|BOLD:AAC7040
Pachycondyla villosa|ID-Unpublished|French Guiana|BOLD:AAC7040
Pachycondyla villosa|ID-Unpublished|French Guiana|BOLD:AAC7040
Pachycondyla villosa|ID-Unpublished|French Guiana|BOLD:AAC7040
Neoponera villosa|ID-Unpublished|Mexico.Campeche|BOLD:AAC7040
Neoponera villosa|ID-Unpublished|Mexico.Campeche|BOLD:AAC7040
Neoponera villosa|ID-Unpublished|Mexico.Campeche|BOLD:AAC7040
Neoponera villosa|ID-Unpublished|Mexico.Campeche|BOLD:AAC7040
Neoponera villosa|ID-Unpublished|Mexico.Campeche|BOLD:AAC7040
Neoponera villosa|BBHYA3544-12|United States.Texas|BOLD:AAC7040
Pachycondyla villosa|ASANA038-06|United States.Texas|BOLD:AAC7040
Pachycondyla villosa|ID-Unpublished|Belize|BOLD:AAC7040
